# Clinical characteristics and treatment-related biomarkers associated with response to high-dose interleukin-2 in metastatic melanoma and renal cell carcinoma: retrospective analysis of an academic community hospital’s experience

**DOI:** 10.1186/s40064-015-0890-1

**Published:** 2015-03-07

**Authors:** Christine Saraceni, Nicole Agostino, Michael J Weiss, Katherine Harris, Suresh Nair

**Affiliations:** Department of Hematology Oncology, Lehigh Valley Health Network, John and Dorothy Morgan Cancer Center, 1240 S Cedar Crest Blvd, Suite 401, Allentown, PA 18103 USA; Lehigh Valley Health Network, Health Systems Research and Innovation, 1628 Chew Street, 3rd Floor, Allentown, PA 18102 USA

**Keywords:** Interleukin-2, Metastatic melanoma, Metastatic renal cell carcinoma, IL-2, Biomarkers, Safety, Response

## Abstract

**Background:**

Immunotherapy in the treatment of metastatic melanoma and renal cell carcinoma can produce durable therapeutic responses, which may improve survival. We aimed to identify clinical characteristics and biomarkers associated with response to high-dose interleukin-2 therapy (IL-2) in patients with metastatic melanoma and renal cell carcinoma treated at an academic community hospital.

**Patients/Methods:**

We retrospectively analyzed clinical variables and biomarkers of 50 consecutive metastatic melanoma or renal cell carcinoma patients treated at our institution with IL-2 during 2004 – 2012. We evaluated clinical characteristics: metastatic sites of disease, prior therapies, number of IL-2 doses per cycle, response duration, autoimmune phenomena, and peak fever, as well as laboratory biomarkers: baseline LDH, platelet nadir, and baseline and highest absolute lymphocyte count (ALC). Survival outcomes were calculated using Kaplan-Meier curves.

**Results:**

Variables differing between responders (clinical benefit group) and non-responders (no clinical benefit group) in metastatic melanoma included platelet nadir during treatment (p = 0.015), autoimmune phenomena (p = 0.049), and in renal cell carcinoma, platelet nadir (p = 0.026). There were no significant differences between number of doses of IL-2 received per cycle and response in either cancer subtype. Clinical benefit occurred in 25% of patients (9/36) when IL-2 was given as first-line therapy. Median overall survival for the clinical benefit group from the initiation of IL-2 to death or last follow-up was 61 months versus 17 months for the no clinical benefit group (p < 0.001) for metastatic melanoma. In renal cell carcinoma overall survival for clinical benefit patients was 48 months versus 17 months. No treatment-related deaths occurred.

**Conclusions:**

High-dose IL-2 can be safely administered by an experienced team in a non–intensive care oncology unit. The clinical benefit group developed autoimmune phenomena (melanoma patients), lower platelet nadir, and on average, received the same number of IL-2 doses as the no clinical benefit group, suggesting a response relationship to the patient’s baseline immune status. Further investigation of immune predictors of response may be useful to select appropriate patients for this therapy.

## Introduction

As the incidence of cutaneous melanoma has risen incrementally in the United States, it has become a focus of public health concern. In 2014, an estimated 76,100 persons will be diagnosed with melanoma, resulting in 9,710 estimated deaths (Cancer Facts and Figures 2014). Kidney and renal pelvis cancers accounted for 63,920 new cases with 13,860 deaths (Cancer Facts and Figures 2014). Survival rates are largely dependent on the stage of disease at initial diagnosis. Prior to 2011, treatment options had been limited and, overall, disappointing for stage IV melanoma with a median survival of less than 1 year (Agarwala 2009). Although the advent of multi-targeted tyrosine kinase inhibitors has significantly improved survival in patients with metastatic renal cell carcinoma (mRCC), the median overall survival during the time period of our study was less than 24 months (Choueiri et al. 2007).

A new paradigm for primary systemic treatment for metastatic melanoma has shifted toward immunotherapy, including IL-2 and anti-CTLA4 monoclonal antibody (ipilimumab) as well as programmed cell death 1 antibody (anti-PD1), and molecularly-targeted therapies (BRAF or MEK inhibitors). Current MM and mRCC treatments are limited in curative potential and are generally palliative. High-dose interleukin-2 (IL-2) is FDA-approved for the treatment of MM and mRCC and is the only potentially curative therapy that exists for both of these diseases. IL-2 immunotherapy, when used for treatment of mRCC, has demonstrated an overall response rate (ORR) of approximately 14%, with 5% of responders having a complete response (CR) (Fyfe et al. 1995). When used in the treatment of MM the ORR is 17%, with 6 – 7% of patients achieving a CR (Rosenberg et al. 1994). Currently there is no way to determine prior to the initiation of therapy which patients will experience complete response and which patients are most likely to benefit from treatment. Herein we detail our single-institutional experience at Lehigh Valley Health Network in a retrospective analysis of 50 consecutive patients with MM or mRCC treated with high-dose IL-2 from August 2004 – August 2012. Our aim was to compare the subset of patients who derived clinical benefit to those patients without apparent clinical benefit as determined by overall survival in months from the initiation of IL-2 therapy. This comparison can be used to better delineate patient characteristics and report patterns in IL-2–treated patients that may be correlated to durable responses as well as aid in selecting patients who are most likely to respond to IL-2 therapy. Our exploratory analyses included pre-treatment and treatment-related patient factors that may be associated with treatment response. Our observations will add to the growing body of knowledge in targeted, personalized cancer care in MM and mRCC. Additionally, we demonstrate that this treatment, when performed under a controlled environment, can be safely administered in an academic community hospital setting. Selection of therapy must account for patient characteristics, toxicities and clinical endpoints. The aim of our study was to better define prognostic variables with response to IL-2 in the metastatic setting and to compare specific characteristics of the clinical benefit group versus those without clinical benefit.

## Patients and methods

### Study design and patient selection

A retrospective analysis was performed on a cohort of 50 consecutive patients with MM or mRCC treated with high-dose IL-2 between August 2004 and August 2012 at Lehigh Valley Health Network, Allentown, Pennsylvania. Our aim was to review our experience in using IL-2 in MM and mRCC patients and to report patterns in IL-2 treated patients who experienced significant survival benefit of >1 year. Evaluation of responses was based on RECIST criteria (Eisenhauer et al. 2009). Patients with oligometastatic stable disease or partial response at the end of therapy were considered for metastectomy on the basis of clinical judgment. Patients rendered disease-free either by IL-2 alone or IL-2 plus metastectomy or patients with durable partial response for >1 year were considered to have “clinical benefit.” Within the clinical benefit group, 2 of the patients had mRCC and 8 had MM.

We additionally report a safety and toxicity analysis of IL-2 therapy when used in an academic community hospital setting. Absolute inclusion criteria for IL-2 treatment included histologically confirmed MM or mRCC (stage IV) based on the tumor, node, metastasis (TMN) criteria, Eastern Cooperative Oncology Group (ECOG) performance score of 0 – 1, magnetic resonance imaging or contrast computed tomography of the brain negative for metastases or with small, asymptomatic or treated metastases, liver function, thyroid stimulating hormone and comprehensive metabolic profile within normal limits, creatinine ≤ 1.5 mg/dL, absolute neutrophil count ≥1500, platelet ≥1000, normal cardiac function as measured by stress test and ejection fraction >50% on echocardiography or MUGA scan, pulmonary function tests within normal limits to 75% of predicted values for age, no ischemic changes on electrocardiogram. Other relative criteria included age <50 and no or minimal visceral disease (exceptions made on case by case basis). In our clinical benefit group, none of the patients had documented brain metastases prior to IL-2 treatment. In the entire cohort, none of the patients had “active” brain metastases prior to IL-2 therapy.

### Ethics statement

The study was approved by Lehigh Valley Health Network’s Institutional Review Board (IRB). All patient records and information was anonymized and de-identified prior to analysis.

### Data collection

Data were extracted from the medical record and included age, gender, disease subtype (melanoma or renal cell carcinoma), time from first day of IL-2 treatment to death, time from stage IV diagnosis to death, metastatic sites, baseline LDH, total number of IL-2 doses received, total number of cycles of IL-2, average dose per cycle, duration of response in months, autoimmune phenomena (hypothyroidism, vitiligo, neuropathy), peak fever, intra-cycle platelet nadir, baseline absolute lymphocytosis (ALC) at the start of cycle 1, highest ALC (average peak ALC across all cycles reported).

### Toxicity

Toxicities were graded for each patient. In this study, only grades 3 and 4 toxicities were reported, as defined by the NCI Common Terminology Criteria for Adverse Events 3.0 (Trotti et al. 2003).

### Treatment

Treatment with HD IL-2 was given in a telemetry-monitored, non–intensive care, specialized oncology unit. HD IL-2 was delivered intravenously: 600,000 units/kg over 15 minutes every 8 hours for a maximum of 14 doses per cycle in accordance with strictly written protocols and NCI guidelines for administration of HD IL-2. Continuous cardiac monitoring occurred throughout the duration of hospitalization. Hemodynamics were assessed every 4 hours and every 30 minutes after IL-2 infusion until a dose-limiting factor precluded further treatments. Patients were closely monitored for side effects and full doses were held or administered at 8-hour intervals on the basis of the presence or absence of relative and absolute criteria as defined by published and institutional guidelines (Table 1). After completion of a cycle of treatment (cycle 1), patients were re-evaluated and admitted in 14 days for the next cycle (cycle 2), administered in the same fashion (2 cycles constituting one course of treatment). Re-staging occurred 6 weeks after every second cycle of IL-2; stable or responding patients were eligible for a total of 3 courses of treatment (6 cycles). Patients with evidence of tumor regression or stable disease would then proceed to additional courses of treatment—no sooner than 6 weeks but no later than 8 weeks—following hospital discharge from the prior course. Treatment was discontinued in patients with evidence of progressive disease or untoward toxicity.

**Table 1:** **Institutional guidelines - relative and absolute criteria for discontinuation of IL-2 therapy**

**System**	**Relative criteria**	**Absolute criteria**
Cardiac	Sinus tachycardia, HR 120 – 130	Sinus tachycardia, HR >130
		EKG Ischemia
		Atrial Fibrillation
		Frequent PVCs, bigeminy, ventricular arrhythmia
		Elevated cardiac enzymes
Dermatologic		Moist desquamation
Gastrointestinal	Diarrhea >1000cc/shift	Refractory vomiting
		Unrelenting abdominal pain
	Abdominal distension	
Hemodynamic		Hypotension requiring pressors
Hemorrhage	Guiac-positive stool, sputum or emesis	Frank blood in stools, sputum or emesis
	Platelets 30,000 – 50,000/mm^3^	
		Platelets < 30,000/mm^3^
Infectious		Strong clinical suspicion or documented
Neurologic		Mental status changes
		Disorientation, vivid dreams, emotional lability
Pulmonary	Resting dyspnea	>4 L oxygen needed for saturations >95%
	Crackles present in >1/3 but <1/2 chest	Crackles present in >1/2 chest
		40% oxygen mask for saturations >95%
	3 – 4 L oxygen for saturations > 95%	
		Intubation
Renal	Urine output 80 – 160 mL/8 hour	Urine <80 mL/8 hour
		Urine < 10 mL/hour
		Creatinine ≥ 3 mg/dL
	Urine 10 – 20 mL/hour	
	Creatinine 2.5 – 2.9 mg/dL	
Weight Gain	15% over baseline	

In the occurrence of any relative criteria the dose is held, corrective action taken and patient re-assessed at next scheduled time. Discontinue therapy if ≥ 3 relative criteria occur or for any one absolute criteria. Discontinue therapy if pressors are needed for hypotension, neurological changes or the scheduled dose has been held on 3 occasions.

HR = heart rate, EKG = electrocardiogram, PVC = premature ventricular contraction).

### Response evaluation

Re-staging studies—including CT scans of the chest, abdomen and pelvis and (where indicated) magnetic resonance imaging (MRI) of the brain—were performed at regular intervals (in general, after 2 cycles or within 2 months from the initiation of therapy).

### Statistical analysis

Descriptive statistics including means, standard deviations, frequencies and percentages were calculated for the clinical benefit and no clinical benefit groups. Continuous variables were compared using Independent Student’s t-tests and Mann–Whitney *U*. They are presented as mean ± standard deviation and median (interquartile range), respectively. Categorical data were compared using Chi-square tests and Fisher’s Exact Tests. Categorical data are presented as frequency (percent).

Overall survival was plotted on a Kaplan-Meier Curve. Differences in group median survival were calculated with the Log-Rank Test. Overall survival was calculated from the date of initiation of IL-2 to death or last follow-up by study closure.

All analyses were conducted using IBM SPSS Statistics for Windows, version 21.0 (IBM Corp., Armonk, NY) and MedCalc for Windows, version 13.0.1.0 (MedCalc Software, Ostend, Belgium).

## Results

We identified 10 patients (2 mRCC and 8 MM) who responded to HD IL-2 therapy (clinical benefit group) and 40 who were non-responders (no clinical benefit group). Fifty consecutive patients were analyzed and included 13 with renal cell carcinoma and 37 with melanoma. There were no significant differences in age, gender, prior treatments, ulceration or BRAF mutational status in melanoma patients, sites of metastatic disease or pretreatment laboratory values including baseline ALC and LDH between the 2 groups. Demographic data can be found in Tables 2 and 3.

**Table 2:** **Baseline characteristics renal cell carcinoma† fisher’s exact test (reported as frequency (percent))**

	**Clinical benefit (n=2)**	**No clinical benefit (n=11)**	**p-value**
Age	47.0 (40.0)	53.0 (46.0-54.0)	.641*
Male	2 (100%)	8 (72.7%)	1.0^†^
Baseline ALC	1.55 (1.30)	1.40 (0.80-1.70)	.641*
LDH	277.5 (158.0)	209.0 (151.0-286.0)	.641*
Prior Treatments	1/2 (50%)	6/11 (54.5%)	1.0^†^
Metastatic Subsets			
Distant Nodes	1 (50%)	2 (18.1%)	.423^†^
Lung +/− Distant Nodes	0	5 (45.5%)	.487^†^
Visceral	1 (50%)	4 (36.4%)	1.0^†^

*Mann–Whitney *U* (reported as median (interquartile range)).

ALC: Absolute Lymphocyte Count.

LDH: Lactate Dehydrogenase.

**Table 3:** **Baseline characteristics metastatic melanoma**

	**Clinical benefit (n=8)**	**No clinical benefit (n=29)**	**P-value**
Age	54.5 (49.0-59.25)	56.0 (43.5-64.5)	.986*
Male	8 (100%)	22 (75.9%)	.308^†^
BRAF Mutated	2/5 (40%)	9/19 (47.4%)	1.00^†^
Baseline ALC	1.50 (0.975-1.775)	1.30 (0.95-1.80)	.842*
LDH	203.5 (164.0-415.75)	244.0 (187.0-542.5)	.251*
Ulceration	2/6 (33.3%)	6/20 (30.0%)	1.00^†^
Prior Treatments	0/8 (0%)	7/29 (24.1%)	0.318^†^
Metastatic Subsets			
IVA	2 (25%)	4 (13.8%)	.591^†^
IVB	1 (12.5%)	6 (20.7%)	1.0^†^
IVC	5 (62.5%)	19 (65.5%)	1.0^†^

^†^Fisher’s Exact test (reported as frequency(percent)).

* Mann–Whitney *U* (reported as median (interquartile range)).

ALC: Absolute Lymphocyte Count.

LDH: Lactate Dehydrogenase.

In our cohort, we defined 10 patients with clinical benefit and compared selected attributes of these patients to those without apparent clinical benefit to identify characteristics in MM and mRCC patients that may be associated with response to immunotherapy. A small subset of our cohort received prior therapies before referral to our IL-2 center (14/50 patients) in the stage IV setting. Within the mRCC group, more than half of the patients (7/13) received other treatments prior to IL-2 while only 7/37 of the patients with MM received prior treatments. Renal cell patients receiving prior treatment included one who received interferon/bevacizumab on a clinical trial and 6 who received targeted therapies (sorafenib, sunitinib, temsirolimus, everolimus). In our melanoma population, 2 received chemotherapy alone (carboplatin/paclitaxel or temozolomide), 2 received chemotherapy and targeted therapy, and 3 received melanoma vaccines on clinical trials. Of the 14 patients who received other therapies prior to IL-2, only one patient was within the clinical benefit group (mRCC patient). The group of 36 patients who received IL-2 as first-line treatment for metastatic disease had 9 patients within the clinical benefit group (25%). Although there appeared to be a difference between prior therapies and response to IL-2 therapy in our cohort, it did not reach statistical significance in either cancer subtype (Tables 2 and 3) To date, 6/50 patients (12%) remain disease-free and in complete remission. Two of these patients were treated with IL-2 alone and 4 received IL-2 followed by metastectomy. Three patients with partial or complete response had eventual disease progression and died. One patient who relapsed at year 3 is now on BRAF/MEK-inhibition.

Treatment and outcome data can be found in Tables 4 and 5. Patients in the clinical benefit group for MM were more likely to receive a greater number of HD IL-2 doses (33.0 versus 18.0, p =0.001) and a greater number of total cycles (6.0 versus 2.0, p <0.001) than those in the no clinical benefit group. In addition, the clinical benefit group had a longer duration of therapy (26.0 versus 1 month, p <0.001). The no clinical benefit group had a higher median platelet nadir (80.0 (69.5 – 133.0)) than the clinical benefit group (50.0 (37.25 – 93.0)) p = 0.015. The clinical benefit group showed a higher frequency of autoimmune phenomenon (p = 0.049). For mRCC, the clinical benefit group received a greater number of IL-2 doses and total cycles (p <0.026) and had a lower platelet nadir.

**Table 4:** **Outcomes metastatic melanoma**

	**Clinical benefit (n=8)**	**No clinical benefit (n=29)**	**p-value**
Total Doses	33.0 (29.0 – 41.75)	18.0 (12.5 – 28.0)	.001*
Total Cycles	6.0 (5.0 – 6.0)	2.0 (2.0 – 4.0)	<.001*
Doses per Cycle	6.4 (5.33 – 6.96)	7.0 (5.42 – 7.38)	.502*
Duration (months)	26.0 (18.25 – 56.25)	1.0 (0 – 4.0)	<.001*
Peak Fever (Celsius)	39.7 (38.5 – 40.3)	39.1 (38.4 – 39.3)	.158*
Peak ALC	3.75 (2.85 – 5.425)	2.9 (1.9 – 4.05)	.137*
Platelets	50.0 (37.25 – 93.0)	80.0 (69.5 – 133.0)	.015*
Autoimmune phenomenon	4 (50.0%)	4 (13.8%)	.049^†^
Any Toxicity	5 (62.5%)	9 (31.0%)	.215^†^
None	3 (37.5%)	20 (68.9%)	.215^†^
Grade 3	3 (37.5%)	3 (10.3%)	.101^†^
Grade 4	2 (25.0%)	6 (20.7%)	.1.0^†^
Grade 5	--	--	--
Survived	6 (75.0%)	7 (24.1%)	.006^†^
Survival Months	61.0	17.0	.001^¥^

^†^Fisher’s Exact test (reported as frequency (percent)).

*Mann–Whitney *U* (reported as median (interquartile range)).

^¥^Log-Rank Test (reported as median, censored data included).

ALC: Absolute Lymphocyte Count.

**Table 5:** **Outcomes renal cell carcinoma**

	**Clinical benefit (n=2)**	**No clinical benefit (n=11)**	**p-value**
Total Doses	46.5 (38.0)	14.0 (10.0 – 22.0)	<.026*
Total Cycles	6.0 (6.0)	2.0 (2.0 – 4.0)	<.026*
Doses per Cycle	7.75 (6.33)	5.5 (5.0 – 6.75)	.231*
Duration (months)	28.0 (23.0)	2.0 (0 – 4.5)	<.026*
Peak Fever (Celsius)	39.3 (39.1 – 39.6)	38.9 (38.7 – 39.8)	.641*
Peak ALC	5.3 (5.1)	3.4 (2.0 – 4.6)	.103*
Platelets	49.0 (33.0)	109.0 (88.0 – 146.0)	.026*
Autoimmune phenomenon	1 (50.0%)	1 (9.1%)	.295^†^
Any Toxicity	2 (100%)	3 (27.3%)	.128^†^
None	0	8 (72.7%)	.128^†^
Grade 3	2 (100%)	2 (18.2%)	.077^†^
Grade 4	0	1 (9.1%)	.1.0^†^
Grade 5	--	--	--
Survived	1 (50.0%)	6 (54.5%)	1.0^†^
Survival Months	48.0	17.0	.231^¥^

^†^Fisher’s Exact test (reported as frequency (percent)).

*Mann–Whitney *U* (reported as median (interquartile range)).

^¥^Log-Rank Test (reported as median, censored data included).

ALC: Absolute Lymphocyte Count.

**Figure 1:**
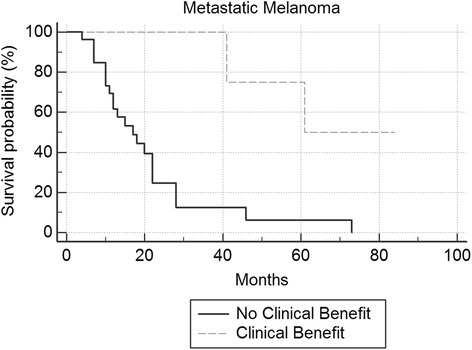
**Kaplan Meier survival curve metastatic melanoma.**

**Figure 2:**
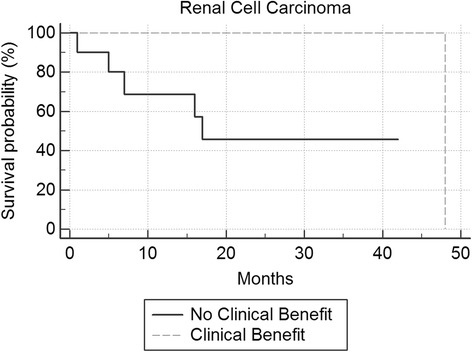
**Kaplan Meier survival curve renal cell carcinoma.**

Seventy-five percent (6 patients) in the MM clinical benefit groups versus 24.1% (7 patients) in the no clinical benefit group were alive at study closure (p = 0.006). For mRCC patients, one in the clinical benefit group and 6 in the no clinical benefit group were alive at study closure. Median overall survival for the entire cohort was 22 months (p = 0.001) for metastatic melanoma and 48 months (p = 0.231) for mRCC. For MM, the median overall survival was 61 months in the clinical benefit group versus 17 months in the no clinical benefit group (p = 0.001) (Figure 1). For mRCC, median survival was 48 months in the clinical benefit group versus 17 months in the no clinical benefit group (Figure 2).

## Discussion

MM and mRCC portend an overall poor prognosis. The 5-year survival rates for mRCC and MM are 12% and 15%, respectively (American Cancer Society 2014). Moreover, the incidence of melanoma has been steadily increasing over the past 30 years (Howlader et al. 1975). For MM, the efficacy of traditional chemotherapeutic agents is mediocre (Trotti et al. 2003). For this reason, improved therapies are needed. Our general treatment philosophy for stage IV disease is immunotherapy—first-line in suitable candidates—followed by targeted therapy and chemotherapy as later options. This is aligned with the 2013 Society for Immunotherapy of Cancer Consensus statement (Kaufman et al. 2013). The consensus panel has recognized several systemic therapies for unresectable stage IV melanoma, including high-dose IL-2, ipilimumab and, in BRAF-mutated tumors, vemurafenib, dabrafenib (BRAF inhibitors) and trametinib (MEK inhibitor), as well as referral for ongoing clinical trials. However, IL-2 immunotherapy has emerged as first-line therapy for stage IV melanoma in patients with good performance status and eligibility per institutional guidelines. In patients with BRAF-mutated melanoma and good performance status, a BRAF inhibitor is reserved for unequivocal evidence of disease progression after immunotherapy. Evaluation for surgical resection of metastases is advisable for all patients, pre- and post-immunotherapy treatment (Kaufman et al. 2013). We demonstrate that IL-2 can be safely administered at centers with teams experienced in the delivery of treatment and management of side effects and toxicities. Furthermore, in carefully selected patients, IL-2 immunotherapy can produce durable therapeutic responses including prolonged disease-free survival and, in some instances, cure.

We identified several prognostic indicators of response to IL-2 treatment. We found for both MM and mRCC that the number of doses per cycle was not significant. However, total doses and cycles received throughout the entire course of treatment was significant (Tables 4 and 5). The most provocative finding is the trend toward greater clinical efficacy of high-dose IL-2 in the first-line setting (25% of patients in clinical benefit group) as opposed to later-line therapy, despite comparable performance status for both groups. Our analyses did not reveal statistical significance although our study most likely is not powered to sufficiently detect true differences.

Our observations suggest a relationship between degree of thrombocytopenia and clinical response to IL-2 treatment. A few other studies have evaluated the relationship between platelet nadir and response to IL-2 therapy with conflicting results (American Cancer Society 2014; Rosenberg et al. 1998; Klapper et al. 2008; MacFarlane et al. 1995; Royal et al. 1996; Bael et al. 2004). Some studies (American Cancer Society 2014; Rosenberg et al. 1998; MacFarlane et al. 1995) have shown no significant association between degree of thrombocytopenia and response while others (Klapper et al. 2008; Royal et al. 1996; Bael et al. 2004) have demonstrated an association between platelet nadir and response. The precise mechanism of development of marked thrombocytopenia in association with response to IL-2 has not been conclusively elucidated. Proposed mechanisms include more adept platelet activation through the inflammatory cascade or mediation of an antibody-producing immune response (MacFarlane et al. 1995; Bael et al. 2004). The latter has been suggested as platelets share common antigens with melanoma tumor cells including glycoprotein IIb/IIIa, CD63 and vitronectin receptor α_V_β_3._ Antibody production against these common antigens may produce an autoimmune thrombocytopenia and concurrent anti-tumor effect (Bael et al. 2004).

Autoimmune phenomena are a well-documented consequence of immunotherapy (Phan et al. 2001; Rosenberg and White 1996; Krouse et al. 1996; Hodi et al. 2010) and have been correlated with survival benefit (Phan et al. 2001; Rosenberg and White 1996; Krouse et al. 1996). Immunoregulatory mechanisms underlying treatment-induced autoimmunity to self (disruption of immunologic tolerance to normal antigens) and the anti-tumor response is an active area of current research (Jiang and Chess 2006). These mechanisms have not been fully elucidated. In our study, development of autoimmune phenomena in metastatic melanoma was associated with response to IL-2.

IL-2 treatment-related toxicities, including systemic capillary leak syndrome, a cascade of plasma extravasation and vascular collapse, limit its delivery in clinical practice. In our cohort, serious toxicities included gastrointestinal bleeding in 2 patients (1 patient with a bleeding gastric metastasis and the other patient with a duodenal ulcer bleed after cycle 5 of treatment), acute pancreatitis in one patient secondary to a pancreatic metastasis, venous thrombosis in 2 patients (1 upper extremity DVT and 1 lower extremity DVT), and arrhythmia (1 patient with paroxysmal atrial fibrillation and 2 patients with non-sustained ventricular tachycardia). In addition, one patient developed grade-3 delayed autoimmune angioedema and uticaria. Serious grade-4 toxicities included acute renal failure (1), hepatotoxicity (1), hypotensive shock (1), congestive heart failure/ pleural effusion (1). Among these, there were 3 intensive care unit (ICU) transfers. Overall, the 3 ICU transfers were able to be managed with aggressive medical measures. The other patients had uneventful recovery from their toxicities with supportive care. There were no treatment-related deaths. An interesting observation was the absence of any peripherally inserted central catheter (PICC) line-related infections or overt bacteremia despite 173 admissions with PICC-line placement for IL-2 therapy.

Limitations to our study include a small cohort of patients included in a retrospective analysis. This is particularly true in our mRCC subset where statistical analysis would likely be more robust with a larger sample size. There may be differences in responding patients although our study may not be powered to detect such differences. Another limitation of our study is incomplete data on both BRAF mutational status and ulceration in melanoma patients. In the early study period, BRAF mutation status was not routinely tested and targeted therapies had not received FDA approval. Both BRAF and ulceration are adverse prognosticators and may potentially bias the outcome measures. Prior studies, including prospective trials (Phan et al. 2001; Klapper et al. 2008; Royal et al. 1996; Tarhini et al. 2007), have validated our observations on a larger scale. Additionally, our survival data are incomplete as many patients are still alive after the study closure, which may underestimate the survival benefit in clinical responders. Because our study represents a small sample of patients, findings should be evaluated in this context. Survival in our mRCC subset should be interpreted in the context of a limited sample size. The provocative OS of 48 months in our 10 mRCC patients treated with IL-2 may represent the controversial observation reported by others (Birkhauser et al. 2013) that high-dose IL-2 may improve the duration of response to salvage targeted therapy used at disease progression.

In summary, factors significantly associated with response to IL-2 from our single institutional experience in treating patients with MM or mRCC include development of autoimmune phenomena including secondary hypothyroidism, vitiligo and neuropathy in metastatic melanoma and lower platelet nadir. Patients treated with IL-2 as first-line therapy had a higher chance of clinical benefit. The number of doses per cycle did not influence clinical benefit, and these authors feel that it is unwise to push patients to receive a higher number of doses in the face of significant toxicity. We could not find a statistical significance to baseline LDH and clinical benefit, as has been reported by others (Davar et al. 2014). This may be due to our exclusion of patients with very high LDH levels with active visceral metastases, as the LDH levels were somewhat homogenous for our treated group. We also demonstrate that IL-2 can be safely and effectively administered in a community hospital setting by using a systematic approach, which includes an experienced health-care team that is knowledgeable in delivering treatment and in managing treatment-related side effects and toxicities. The delivery of IL-2 in a more patient-friendly, non-ICU medical/surgical unit and treating appropriate patients in the first-line setting may improve the value and cost-effectiveness of this curative approach. In our institution’s experience, treating appropriate patients in the first-line setting demonstrated a 25% clinical benefit, leading to a median survival of greater than 5 years in metastatic melanoma (Figure 1), and 48 months in mRCC (Figure 2) although our findings will need to be validated in larger, prospective studies.
